# The New Cure of Consumption by Its Own Virus

**Published:** 1892-03

**Authors:** 


THE NEW CURE OF CONSUMPTION BY ITS OWN VIRUS. Illustrated by Numerous Cases. By J. Compton Burnett, M. D. Second edition. Revised and enlarged. "Ubi morbus ibi remedium." Philadelphia: Bœricke & Tafel, 1892. Price, 90 cents.

Dr. Burnett's little volume is out in a second edition within a year. Our readers have no doubt read the first edition, and know the tendency of the little work. In this edition he has added some sixty pages of reading matter—his latest experience. Let him *prove* his Bacillinum, and then we can all use it when indicated. As it is, his Bacillinum is no better than Koch's Tubercullin, and we know the fate of the latter by this time. W. S.

